# Phylogeny and evolution of streptophyte algae

**DOI:** 10.1093/aob/mcae091

**Published:** 2024-06-04

**Authors:** Maaike J Bierenbroodspot, Thomas Pröschold, Janine M R Fürst-Jansen, Sophie de Vries, Iker Irisarri, Tatyana Darienko, Jan de Vries

**Affiliations:** Department of Applied Bioinformatics, Institute for Microbiology and Genetics, University of Goettingen, Goldschmidtstraße 1, 37077 Goettingen, Germany; Department of Applied Bioinformatics, Institute for Microbiology and Genetics, University of Goettingen, Goldschmidtstraße 1, 37077 Goettingen, Germany; Research Department for Limnology, University of Innsbruck, Mondseestr. 9, 5310 Mondsee, Austria; Department of Applied Bioinformatics, Institute for Microbiology and Genetics, University of Goettingen, Goldschmidtstraße 1, 37077 Goettingen, Germany; Department of Applied Bioinformatics, Institute for Microbiology and Genetics, University of Goettingen, Goldschmidtstraße 1, 37077 Goettingen, Germany; Section of Phylogenomics, Centre for Molecular Biodiversity Research, Leibniz Institute for the Analysis of Biodiversity Change (LIB), Museum of Nature, Hamburg, Martin-Luther-King Platz 3, 20146 Hamburg, Germany; Department of Applied Bioinformatics, Institute for Microbiology and Genetics, University of Goettingen, Goldschmidtstraße 1, 37077 Goettingen, Germany; Department of Experimental Phycology and Culture Collection of Algae, Albrecht-von-Haller-Institute for Plant Sciences, University of Goettingen, Nikolausberger Weg 18, 37073 Goettingen, Germany; Department of Applied Bioinformatics, Institute for Microbiology and Genetics, University of Goettingen, Goldschmidtstraße 1, 37077 Goettingen, Germany; Campus Institute Data Science (CIDAS), University of Goettingen, Goldschmidstraße 1, 37077 Goettingen, Germany; Department of Applied Bioinformatics, Goettingen Center for Molecular Biosciences (GZMB), University of Goettingen, Goldschmidtstraße 1, 37077 Goettingen, Germany

**Keywords:** streptophyte algae, evolution, phylogenetics, phylogenomics, terrestrialization, green lineage, Chloroplastida

## Abstract

The Streptophyta emerged about a billion years ago. Nowadays, this branch of the green lineage is most famous for one of its clades, the land plants (Embryophyta). Although Embryophyta make up the major share of species numbers in Streptophyta, there is a diversity of probably >5000 species of streptophyte algae that form a paraphyletic grade next to land plants. Here, we focus on the deep divergences that gave rise to the diversity of streptophytes, hence particularly on the streptophyte algae. Phylogenomic efforts have not only clarified the position of streptophyte algae relative to land plants, but recent efforts have also begun to unravel the relationships and major radiations within streptophyte algal diversity. We illustrate how new phylogenomic perspectives have changed our view on the evolutionary emergence of key traits, such as intricate signalling networks that are intertwined with multicellular growth and the chemodiverse hotbed from which they emerged. These traits are key for the biology of land plants but were bequeathed from their algal progenitors.

## INTRODUCTION

Streptophytes, together with chlorophyte and prasinodermatophyte algae (Li *et al.*, 2020*b*), form the clade known as Chloroplastida (Adl *et al.*, 2005). Chloroplastida are one of the three major lineages of primary photosynthetic eukaryotes (Archaeplastida), whose origin can be traced back to the primary endosymbiotic acquisition of the cyanobacterial progenitor of plastids (Archibald, 2015; Ponce-Toledo *et al.*, 2017; Irisarri *et al.*, 2022) ~2 billion years ago (Strassert *et al.*, 2021). The acquisition of a photosynthetic organelle required substantial genomic and cellular changes, such as the transfer of hundreds to thousands of genes via endosymbiotic gene transfer, the establishment of protein import machineries into the plastid, biochemical coordination, and more (Timmis *et al.*, 2004; Archibald, 2015; de Vries and Archibald, 2017). In recent years, the discovery of two non-photosynthetic lineages that cluster within Archaeplastida, namely Rhodelphidia and Picozoa, have suggested a more complex history for the early evolution of Archaeplastida and of primary plastids (Gawryluk *et al.*, 2019; Schön *et al.*, 2021; Irisarri *et al.*, 2022).

**Fig. 1.**
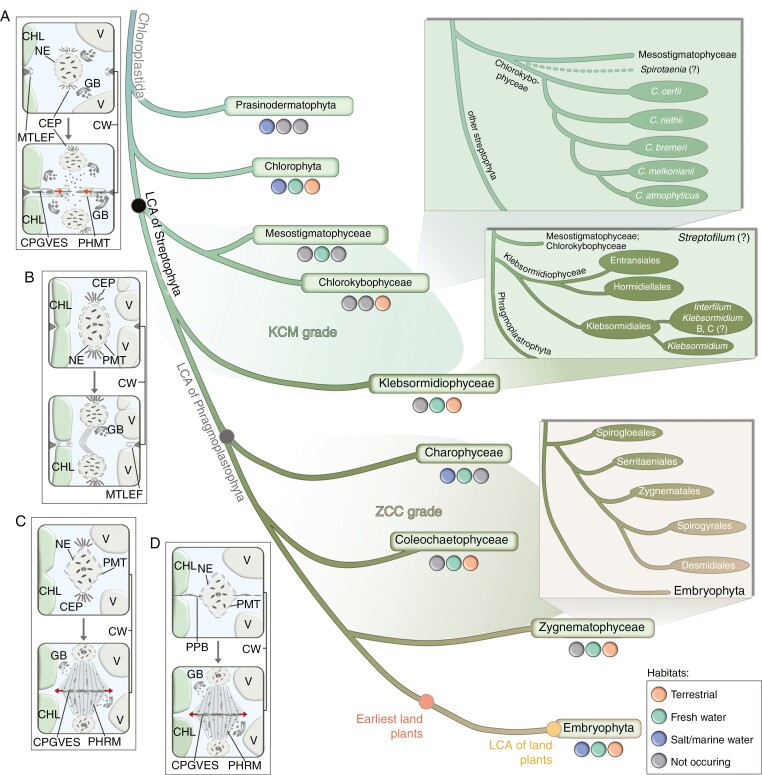
Biodiversity along the phylogeny of the green lineage, with focus on streptophyte algae. A cladogram of the current status of streptophyte relationships from the latest molecular phylogenetic and phylogenomic studies. Cladograms in inset boxes on the right are based on studies by Irisarri *et al.* (2021), Hess *et al.* (2022) and Bierenbroodspot *et al.* (2024). Habitats are derived from Fürst-Jansen *et al.* (2020) and Li *et al.* (2020b). (A) Example of cytokinesis via phycoplast formation in chlorophytes (Ulvophyceae: *Ulothrix*). Prophase and telophase of the closed mitosis is shown. Arrows indicate the formation of a phycoplast. (B) Example of cytokinesis via centripetal cleavage in streptophyte algae (Klebsormidiophyceae: *Klebsormidium*). Prophase and telophase of an open mitosis are shown. (C) Example of cytokinesis via formation of a centrifugal phragmoplast in streptophyte algae (Coleochaetophyceae: *Coleochaete*). Prophase and telophase of an open mitosis are shown. Arrows indicate the formation of an early phragmoplast. (D) Example of cytokinesis in Embryophyta (*Arabidopsis*). Prophase and telophase of an open mitosis are shown. Arrows indicate the formation of an early phragmoplast. Cell division schemata are based on: Floyd *et al.* (1972), Marchant and Pickett‐Heaps (1973), van den Hoek *et al.* (1988), Buschmann and Zachgo (2016), Lokhorst and Star (1985) and Livanos and Muller (2019). Abbreviations: CHL, chloroplast; CPGVES, cell plate of Golgi-derived vesicles; CW, cell wall; CEP, pair of centrioles; GB, Golgi body; MTLEF, microtubules along leading edge of cleavage furrow of plasma membrane; NE, nuclear envelope; PER, perinuclear endoplasmic reticulum; PHMT, phycoplast microtubules; PHRM, phragmoplast microtubule; PMT, perinuclear microtubules; V, vacuole.

The Chloroplastida fist diverged into the Prasinodermatophyta and a clade of progenitors of Chlorophyta plus Streptophyta (Li *et al.*, 2020*b*) (Fig. 1), which consist of several ancient lineages of green algae. Chlorophytic green algae have a diverse range of forms, from microeukaryotes (even pikoeukaryotes) to macrophytes; they dwell in a range of habitats, both when looking at broad categories, such as marine freshwater, and terrestrial habitats, and when considering growth habits, such as epiphytic and even a reversion from phototrophy to heterotrophy (Figueroa-Martinez *et al.*, 2015).

Streptophyta are a species-rich group. This is largely attributable to the high species diversity of flowering plants in Embryophyta, which Darwin coined an ‘abominable mystery’: >450 000 species of angiosperms (Pimm and Joppa, 2015; Corlett, 2016; Zuntini *et al.*, 2024). Indeed, the number of species within the other major lineages of land plants are relatively commonplace: ~9000 mosses (Magill, 2014), 7000 liverworts (Von Konrat *et al.*, 2014), 250 hornworts (Villarreal *et al.*, 2014), slightly >1000 lycophytes (Christenhusz and Byng, 2016), 10 000 ferns (Ranker and Sundue, 2015) and 1000 gymnosperms (Christenhusz *et al.*, 2011). The diversity of Embryophyta has been subject to several phylogenomic investigations, which established a solid framework for the major lineages (Wickett *et al.*, 2014; Puttick *et al.*, 2018; One Thousand Plant Transcriptomes Initiative, 2019). Arguably, the two most stunning revelations, which were recovered in all the studies mentioned, were that bryophytes are likely to be monophyletic and that Zygnematophyceae are the algal sister lineage to land plants (see also Wodniok *et al.*, 2011). But what about the relationships within the algal lineages?

In this article, we take a deep dive into the phylogeny and evolution of streptophyte algae. Given their deep evolutionary splits, there are divergences into order-level lineages of streptophyte algae that pre-date by far the origin of land plants. To tackle this diversity in a systematic fashion, we initially provide an overview of the taxonomy and biodiversity of streptophyte algae. Then we move on to describe how recent advancements in transcriptomic and genomic data generation have paved the way towards a phylogenomic understanding of streptophyte evolution. Finally, we illustrate how phylogenomics perspectives allow us to trace the emergence of key traits, such as molecular adaptations and body plan innovations.

## OVERVIEW OF THE STREPTOPHYTE ALGAL DIVERSITY AND THE CURRENT TAXONOMY

**Fig. 2.**
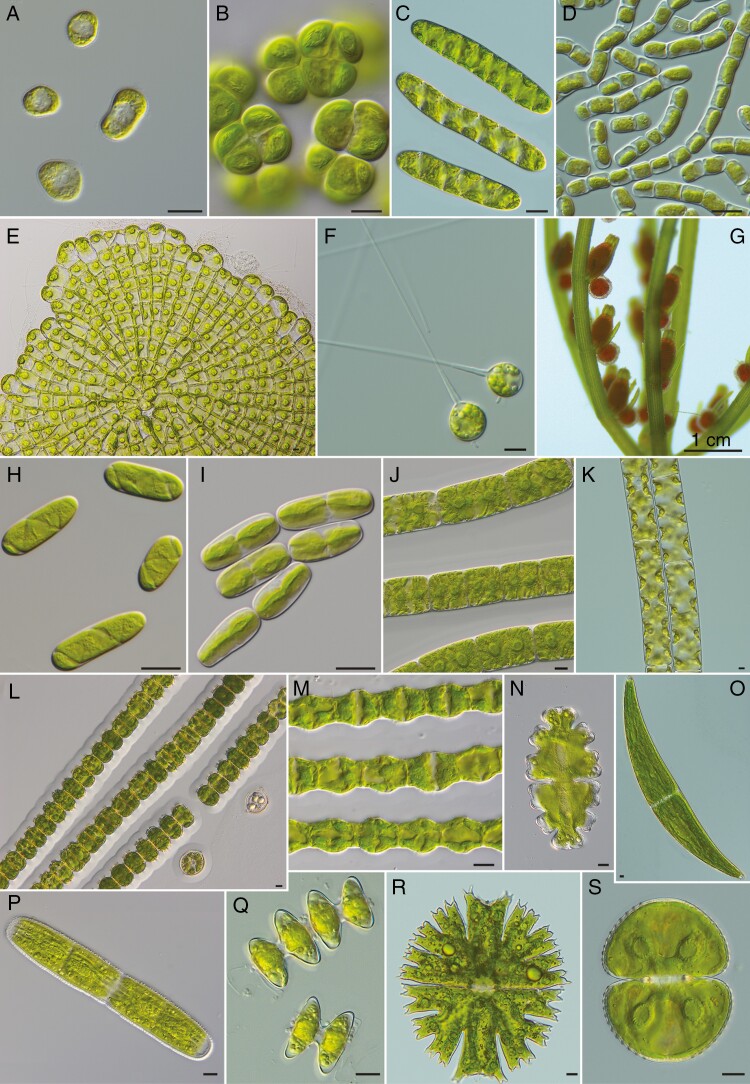
Morphological diversity of streptophytes. Light micrographs for representative species of major streptophyte algal lineages. (A) *Mesostigma viride* (SAG 50-1). (B) *Chlorokybus cerffii* (SAG 34.98). (C) *Spirotaenia condensata* (MZCH 312). (D) *Klebsormidium nitens* (SAG 335-1a). (E) *Coleochaete scutata* (SAG 50.90). (F) *Chaetosphaeridium* sp. (strain Cimino 7c2). (G) *Chara* sp. (field material). (H) *Spirogloea muscicola* (CCAC 0214). (I) *Mesotaenium endlicherianum* (SAG 12.97). (J) *Zygnema circumcarinatum* (SAG 698-1b). (K) *Spirogyra pratensis* (MZCH 10213). (L) *Desmidium grevillei* (SAG 637-1a). (M) *Bambusina brebissonii* (SAG 20.82). (N) *Euastrum oblongum* (SAG 147.80). (O) *Closterium ehrenbergii* (SAG 611-11). (P) *Penium margaritaceum* (SAG 2640). (Q) *Staurastrum punctulatum* (SAG 19.83). (R) *Micrasterias fimbriata* (SAG 162.80). (S) *Cosmarium botrytis* (SAG 136.80). Scale bar: 10 µm (except G).

The Streptophyta (sometimes still called Charophyta) are a monophyletic group of organisms comprising land plants (embryophytes) and the streptophyte algae. Streptophyte algae are morphologically diverse (Fig. 2) and are represented by six main lineages: the earlier-diverging (1) Mesostigmatophyceae and (2) Chlorokybophyceae, (3) Klebsormidiophyceae, and the later-diverging Phragmoplastophyta, formed by (4) Charophyceae, (5) Coleochaetophyceae and (6) Zygnematophyceae, which are the sister group of Embyophyta that are also nested within Phragmoplastophyta (Fig. 1A; Wodniok *et al.*, 2011; Delwiche and Cooper, 2015; Cheng *et al.*, 2019; One Thousand Plant Transcriptomes Initiative, 2019). Although taxonomical and nomenclatorial problems of streptophyte algae are still not completely resolved, there has been major progress in recent years, and overall, there is a solid phylogenomic framework into which streptophyte diversity can be sorted. The present state of the streptophyte algal taxonomy is summarized in Box 1. Six taxonomic classes have been recognized.

### Mesostigmatophyceae

The class Mesostigmatophyceae (Marin & Melkonian, 1999), currently represented by one genus, *Mesostigma* Lauterborn, had originally been assigned to the prasinophytes (Melkonian, 1989) but was later [after progress in its chloroplast-based phylogenetic investigation (Lemieux *et al.*, 2000)] determined to be a streptophyte (Lemieux *et al.*, 2007). This genus contains two species: *Mesostigma viride* Lauterborn and *Mesostigma grande* Korshikov (Table 1). Both species are scaly asymmetrical flagellates that occur in freshwater habitats. *Mesostigma grande* was found only once and therefore declared as uncertain (Guiry, 2024).

**Table 1.** Numbers of species among streptophyte algae.

Class	Number of genera	Number of species	Habitat
Mesostigmatophyceae	1	2	Freshwater
Chlorokybophyceae	1	5	Terrestrial
Klebsormidiophyceae	6	42	Freshwater, terrestrial
Coleochaetophyceae	2	22	Freshwater
Charophyceae	6	395–450	Freshwater–brackish
Zygnematophyceae	>50	4000–13 000	Freshwater, terrestrial

### Chlorokybophyceae

Chlorokybophyceae are exclusively terrestrial organisms with simple package-like morphology that reproduce asexually by asymmetrical, motile zoospores with ultrastructural streptophyte characteristics (Rogers *et al.*, 1980; Lokhorst *et al.*, 1988; Irisarri *et al.*, 2021). Similar to *Mesostigma*, *Chlorokybus* Geitler are uncommon organisms (Geitler, 1942). Currently, a single genus, *Chlorokybus*, is recognized, and it is represented by five cryptic species (Table 1) that were once united under the umbrella name *Chlorokybus atmophyticus* Geitler (Irisarri *et al.*, 2021). Representatives of this genus were found in soils of Europe, Asia and South America. In both Mesostigmatophyceae and Chlorokybophyceae, sexual reproduction is unknown (Lewis and McCourt, 2004). Curiously, molecular phylogenetic analyses have found that the genus *Spirotaenia* Brébisson is closely related to Chlorokybophyceae and Mesostigmatophyceae (Gontcharov and Melkonian, 2004; One Thousand Plant Transcriptomes Initiative, 2019; Irisarri *et al.*, 2021) rather than Zygnematophyceae, as supported by solid morphological features, such as a special type of sexual reproduction (conjugation) and absence of any flagellated stages. A focused study of *Spirotaenia* should clarify this taxonomic conundrum and perhaps reveal overlooked morphological features or evolutionary patterns.

### Klebsormidiophyceae

Klebsormidiophyceae are rich in highly relevant traits for understanding plant terrestrialization. This is astounding, given their ~800-million-year divergence from land plants (for current molecular clocks, see Bierenbroodspot *et al.*, 2024; Bowles *et al.*, 2024). The bodies of Klebsormidiophyceae consist of unbranched uniserial filaments or, rarely, sarcinoid packages (*Streptosarcina* Mikhailyuk & Lukešová and *Interfilum* Chodat), which produce zoospores with streptophyte features (Cook, 2004*b*; Sluiman *et al.*, 2008). The genus *Klebsormidium* P.C.Silva, Mattox & W.H.Blackwell is the type genus of the class and the most prominent organism of this group. Indeed, *Klebsormidium* became a model organism in plant evolutionary biological research, and several of the recent findings on the molecular evolution of key traits discussed later were made through work on *Klebsormidium*. This is not only because *Klebsormidium nitens* (Kützing) Lokhorst was the first streptophyte algal genome to be sequenced (Hori *et al.*, 2014) but because it is resilient and grows well; traits that are apt in the laboratory. Species of *Klebsormidium* are distributed in freshwater and acidic habitats, in addition to many different types of humid habitats in the wider sense around the world. It is one of several algae capable of forming biological soil crusts. Other genera of Klebsormidiophyceae are very rare algae (except for *Interfilum*). Bierenbroodspot *et al.* (2024) split this class into three orders: Klebsormidiales, Hormidiellales and Entransiales. In total, Klebsormidiophyceae includes five genera: *Klebsormidium* (32 species), *Interfilum* (three species), *Hormidiella* M.O.P.Iyengar & Kanthamma (two species), *Streptosarcina* (two species) and *Entransia* E.O.Hughes (two species) (Guiry, 2024) (Table 1). Potentially, the genus *Streptofilum* Mikhailyuk & Lukešová (one species) also belongs to this class, but this is a matter of current research (see below in the section on phylogenomics).

### Charophyceae

The Charophyceae (stoneworts) include freshwater (occasionally brackish) algae with complex macroscopic thalli composed of a main axis with whorled branches characterized by the growth of apical meristematic cells. Sexual reproduction is a specialized oogamous mode, with oogonia and antheridia surrounded by sterile cells. Charophyceae are well represented in the fossil record, with a large diversity extending back to the Silurian (McCourt *et al.*, 2004, 2023; Leliaert *et al.*, 2012). According to McCourt *et al.* (2017), currently six extant genera are recognized, all belonging in the order Charales, family Characeae. Two additional orders and many families and genera are known only from the fossil record (Lu *et al.*, 1996; Feist *et al.*, 2005). Two genera (*Chara* L., *Nitella* C.Agardh) contain >200 described species each, with a third [*Tolypella* (A.Braun) A.Braun] containing several dozen taxa; the remaining genera have only one or a few species (Table 1). Characeae have a worldwide distribution, despite some taxa being endemic or geographically restricted. In general, dioecious taxa are more narrowly distributed or endemic, whereas monoecious taxa are more widely distributed (Proctor, 1980).

### Coleochaetophyceae

Among multicellular streptophytes, the class Coleochaetophyceae are most exciting when it comes to diversity in body plans; they are the Swiss Army Knife of streptophyte algal developmental traits. This class currently includes only two genera: *Chaetosphaeridium* Klebahn and *Coleochaete* Brébisson. *Coleochaete* species can grow not only as bodies of branched filaments, but some species can also develop complex discoid parenchymatous thalli (Graham, 1984). Certain thalli cells bear distinctive sheathed hairs (Printz, 1964), and some species of *Coleochaete* form corticated zygotes that are often retained in the mother plant. The cytokinesis mode, phragmoplast formation and presence of plasmodesmata are similar to those in land plants (Marchant and Pickett‐Heaps, 1973; van den Hoek *et al.*, 1988; Graham *et al.*, 2000; Cook, 2004*a*). The zygote possesses algean, a highly resistant substance that resembles sporopollenin (Delwiche *et al.*, 1989). *Coleochaete* is typically a freshwater organism that can be found growing on aquatic plants, macrophyte algae or stones. Of the 22 recognized *Coleochaete* species (Table 1), only eight are available in public culture collections (Delwiche *et al.*, 2002). The most common species are *Coleochaete scutata* Brébisson, *Coleochaete nitellarum* Jost and *Coleochaete pulvinata* A.Braun.

The position of *Chaetosphaeridium* within Coleochaetophyceae is supported by molecular phylogenetic studies (Karol *et al.*, 2001; Turmel *et al.*, 2002) and by morphological and cytological features, such as the presence of typical sheathed hairs and a similar chloroplast structure (Delwiche *et al.*, 2002; Hall and Delwiche, 2007; Leliaert *et al.*, 2012). *Chaetosphaeridium* consists of many loosely connected globose or flask-shaped cells imbedded in a gelatinous matrix. Vegetative cells bear long, sheathed hairs. Seven species of *Chaetosphaeridium* are described (Table 1), but only two, *Chaetosphaeridium pringsheimii* Klebahn (type species of the genus) and *Chaetosphaeridium globosum* (Nordstedt) Klebahn, are deposited in public culture collections.

### Zygnematophyceae

The Zygnematophyceae, also known as conjugating green algae, are the most species-rich (and morphologically volatile) lineage of streptophyte algae (Leliaert *et al.*, 2012; Hess *et al.*, 2022). Morphologically, representatives of this class are highly diverse, encompassing unicellular coccoid and small colonial forms (‘Desmidiales’ in the traditional sense) and filamentous forms (‘Zygnematales’ in the traditional sense). Sexual reproduction occurs by a unique process of conjugation, involving the fusion of non-motile gametes. The absence of flagellated reproductive stages, plasmodesmata and basal bodies also sets Zygnematophyceae apart from other streptophyte algae. Traditionally, Zygnematophyceae were divided into the Zygnematales and the Desmidiales, based primarily on differences in cell wall structure, but molecular phylogenies have shown that the Zygnematales (characterized by smooth, non-ornamented cell walls) are a paraphyletic assemblage (McCourt *et al.*, 2000; Gontcharov *et al.*, 2003, 2004; Gontcharov and Melkonian, 2004; Gontcharov, 2008; Leliaert *et al.*, 2012). Recently, the Zygnematophyceae were subdivided into five orders based on a phylogenomic analysis (Hess *et al.*, 2022). The orders Spirogloeales, originally erected by Cheng *et al.* (2019), and Desmidiales were confirmed, the order Zygnematales was redefined, Spirogyrales was re-established, and the new order Serritaeniales was proposed. The taxonomic classification at family levels is complex, as exemplified by Guiry (2013), who found many families to be polyphyletic. This prompts for a revised taxonomic effort. The Zygnematophyceae occur in a wide variety of freshwater habitats, such as ephemeral pools, ponds, lakes, marshes, bogs or artificial habitats on every continent around the World. Within a given habitat, species often show a preference for microhabitats. Planktonic species occupy the water column, either permanently as euplankton or temporarily as tychoplankton after being dislodged from the substrate. Relatively few desmid species are truly planktonic. Most conjugating green algae are benthic or periphytic and grow on surfaces or, occasionally, attached to substrates by means of rhizoids or mucilage (Hall and McCourt, 2017). Some Zygnematophyceae could be found in extreme habitats, such on snow and ice [*Ancylonema* Berggren, *Cylindrocystis* Meneghini ex De Bary and *Mesotaenium* Nägeli (Kol, 1968; Procházková *et al.*, 2021; Remias and Procházková, 2023)]. *Cylindrocystis* has also been found in desert crust communities (Lewis and Lewis, 2005; Büdel *et al.*, 2009). The filamentous *Zygogonium* Kützing can be found in habitats that pose a severe challenge to many other streptophytes, including extremely acidic pools and rivers (pH < 3) (e.g. Amaral Zettler *et al.*, 2002), Alpine soils (Stancheva *et al.*, 2014, 2016), or disturbed and acidic mineral soils (Transeau, 1933; Lynn and Brook, 1969; Ettl and Gärtner, 2014). In general, desmids prefer slightly acidic waters (pH 4–7), such as pools in acid peat bogs (Ralfs, 1848; Lenzenweger, 1996, 1997, 1999, 2003).

The Desmidiales (41 genera, >3500 species) are represented by four families: the Closteriaceae, Gonatozygaceae, Peniaceae and Desmidiaceae. The Desmidiaceae are the largest family, with ~36 genera, 3000 species and 12 000 subspecific taxa (Hall and McCourt, 2017). The taxonomic classification is turbulent (Gontcharov and Melkonian, (2010) and at family level needs to be revised by the analysis of more species and genera. As Hess *et al.* (2022) showed, several taxa (e.g. *Mesotaenium* and *Cylindrocystis*) originally assigned to the Desmidiales belong to other orders (Serritaeniales and Zygnematales, respectively). The order Zygnematales traditionally included the families Zygnemataceae and Mesotaeniaceae (Mix, 1972). The family Zygnemataceae include 14 genera and >800 species. The most prominent genus of this family is *Spirogyra* (now Spirogyrales Hess *et al.*, (2022)), with ~300 morphospecies. In contrast, the family Mesotaeniaceae contain only eight genera and ~100 species. Molecular phylogenetic studies indicate that the families of Zygnematales are not monophyletic, and morphological features are often not in correspondence with the phylogeny (McCourt *et al.*, 2000; Gontcharov *et al.*, 2003; Hall *et al.*, 2008*a*).

## EXCITING TIMES FOR PHYLOGENOMICS OF STREPTOPHYTES

The advent of high-throughput sequencing technologies and computational methods paved the way for genome-scaled phylogenomic studies, which now permit the inference of evolutionary relationships among streptophytes with increased precision and accuracy. A phylogenomic backbone of the Streptophyta was established recently (Wickett *et al.*, 2014; Puttick *et al.*, 2018; One Thousand Plant Transcriptomes Initiative, 2019), in which streptophytes consist of five major lineages that branch off successively as (1) Mesostigmatophyceae plus Chlorokybophyceae, (2) Klebsormidiophyceae, (3) Coleochaetophyceae, (4) Charophyceae and (5) the monophylum formed by Zygnematophyceae and land plants (Embryophyta) (Fig. 1). Thus, streptophyte algae form a paraphyletic grade with respect to land plants. Sometimes, streptophyte algal classes are arranged into the KCM grade (Klebsormidiophyceae, Chlorokybophyceae and Mesostigmatophyceae) and the ZCC grade (Zygnematophyceae, Coleochaetophyceae and Charophyceae) (de Vries *et al.*, 2016). This backbone configuration of the streptophyte tree has since been recovered by several independent phylogenomic studies (e.g., Irisarri *et al.*, 2021; Hess *et al.*, 2022; Bierenbroodspot *et al.*, 2024; Bowles *et al.*, 2024). One of the most surprising revelations of recent phylogenomic analyses was that the recovery of Zygnematophyceae as the sister group of Embryophyta, instead of the morphologically more complex Coleochaetophyceae or Charophyceae, as had been proposed earlier based on their more complex multicellular bodies (assumed to be a shared and derived feature) and molecular phylogenies based on only few molecular markers (Graham *et al.*, 2000; Becker and Marin, 2009; Wodniok *et al.*, 2011; Wickett *et al.*, 2014). This result, in addition to the recovery of monophyletic bryophytes (Wickett *et al.*, 2014; Puttick *et al.*, 2018), showcases how a careful analysis of large phylogenomic datasets can resolve difficult evolutionary relationships in the streptophyte tree. In fact, a yet unpublished phylogenomic study including a new species of *Spirogloea* Melkonian (Melkonian *et al.*, 2024) proposed that Spirogloeales (originally recovered as sister to all other Zygnematophyeae; Cheng *et al.*, 2019) might, in fact, be the closest algal sister group to Embryophytes. Should this result pass the test of time, the implications for understanding the evolution of streptophytes are huge. Overall, this and other difficult-to-resolve relationships are often due to fast and ancient radiations supported by rather few molecular synapomorphies and/or confounded by non-phylogenetic noise (e.g. Irisarri *et al.*, 2022). Resolving these recalcitrant nodes is not only important for systematics and taxonomic classifications (Box 1) but is also essential to understand the origin and evolution of key traits in plants.

Beyond the phylogenetic backbone, there is much more to unpack in streptophyte algal diversity and evolution. This is exemplified by several recent phylogenomic analyses within specific streptophyte algal classes. These studies are not only proposing important revisions to streptophyte algal systematics and classification, but also have the power to reveal new insights into the intricate patterns of trait evolution in streptophytes.

Recent phylogenomic studies have confirmed the position of *Mesostigma viride* as a sister to Chlorokybophyceae and *Spirotaenia* sp. (Liang *et al.*, 2020; Wang *et al.*, 2020; Irisarri *et al.*, 2021; Bierenbroodspot *et al.*, 2024). Phylogenomic analyses have also identified five cryptic species in Chlorokybophyceae, with some of the species having diverged ≤76 million years ago (based on molecular clock estimates), about twice as much as between two *Arabidopsis* species (Irisarri *et al.*, 2021). These five cryptic species occur in the absence of observable morphological differences, but transcriptomic data suggest genetic differences, revealed by steady-state differences in gene expression when grown together in the same conditions (Irisarri *et al.*, 2021). An extensive search of metabarcoding data from soil confirmed that *Chlorokybus* is a rare species (Irisarri *et al.*, 2021).

Bierenbroodspot *et al.* (2024) established a three-order system for Klebsormidiophyceae. Molecular clock analyses identified a deep split between Klebsormidiales and the other two orders ~830 million years ago, followed by a split between Entransiales and Hormidiellales ~666 million years ago. Within Klebsormidiales, two issues remain unresolved. Firstly, *Klebsormidium* is not monophyletic, and the type species, *Klebsormidium flaccidum* (Kützing) P.C.Silva, Mattox & W.H.Blackwell (clades B/C), forms a sister group to *Interfilum*, whereas the remaining *Klebsormidium* species (clades D–G) are more distantly related. The second issue concerns the interesting alga *Streptofilum*, which forms filaments and cell packages (Mikhailyuk *et al.*, 2018). The phylogenetic position of *Streptofilum* remains ambiguous. Glass *et al.* (2023), based on plastid-encoded large subunit of RuBisCO (*rbc*L), small-subunit rRNA (SSU) and 44 chloroplast genes, proposed that *Streptofilum* is a separate lineage outside of Klebsormidiophyceae. Instead, Bierenbroodspot *et al.* (2024), relying on analyses of hundreds of nuclear genes, recovered *Streptofilum* within Klebsormidiales, specifically within the *Interfilum* clade. Žárský and Eliáš (2024) reanalysed the dataset of Bierenbroodspot *et al.* (2024) and recovered the same topology as Bierenbroodspot *et al.* (2024) except for the position of *Streptofilum*. This incongruence suggests the need for further investigations into the precise phylogenetic position of *Streptofilum*. Beyond systematics, Bierenbroodspot *et al.* (2024) performed an ancestral character state reconstruction of multicellularity using their time-calibrated phylogenomic tree and found support for the emergence of multicellularity ~1 billion years ago, in the common ancestor of Klebsormidiophyceae and Phragmoplastophyta. Broadly defined multicellularity might even date back to an earlier period (Bowles *et al.*, 2024).

## GENOMIC EXPLORATION OF STREPTOPHYTE ALGAE

Streptophytes are among the first eukaryotes for which a fully sequenced nuclear genome was obtained. In 2000, the flowering model plant *Arabidopsis thaliana* was published (The Arabidopsis Genome Initiative, 2000), following *Saccharomyces cerevisiae* (Goffeau *et al.*, 1996), *Caenorhabditis elegans* (The *C. elegans* Sequencing Consortium, 1998) and *Drosophila melanogaster* (Adams MD, 2000). However, it took another 14 years until the first genome of a streptophyte alga was sequenced.

In 2014, the genome of *Klebsormidium nitens* was published and revealed that a whole battery of genes that were previously considered specific to land plants had already emerged in an ancestor of land plants and Klebsormidiophyceae (Hori *et al.*, 2014). This streptophyte algal ancestor >800 million years ago was likely multicellular (Bierenbroodspot *et al.*, 2024). With this finding, the genomic exploration of early streptophyte evolution began. *Chara braunii* followed next, being the first genome of an algal representative of Phragmoplastophyta (Nishiyama *et al.*, 2018). Then a whole barrage of “firsts” for genomes in key phylogenetic positions followed. The first Zygnematophyceae genomes were *Mesotaenium endlicherianum* Nägeli and *Spirogloea muscicola* (Cheng *et al.*, 2019; the former updated by Dadras *et al.*, 2023*b*), quickly followed by the *Penium margaritaceum* Brébisson ex Ralfs (Jiao *et al.*, 2020). Then the genomes of those streptophytes most divergent from land plants were tackled: *Mesostigma viride* [sequenced by two groups independently (Liang *et al.*, 2020; Wang *et al.*, 2020)] and *Chlorokybus melkonianii* (Wang *et al.*, 2020; a species newly described by Irisarri *et al.*, 2021). The only major lineage of streptophyte algae for which no genome is available while penning these lines are the Coleochaetophyceae.

These studies have revealed a considerable variation in genome size. Take the Zygnematophyceae: *Mesotaenium endlicherianum* (163 Mb) and *Spirogloea muscicola* (174 Mb) have relatively small genomes (Cheng *et al.*, 2019), whereas *Penium margaritaceum* has an estimated haploid genome of 4.7 Gb, and the current genome assembly has 332 786 scaffolds with a cumulative size of 3.661 Gb and an N50 contiguity measure of 116.1 kb (Jiao *et al.*, 2020). With this, the *Penium margaritaceum* genome is twice the size of the macroscopic alga *Chara braunii* (Nishiyama *et al.*, 2018). How did the *Penium* genome become so large? Most of its genome assembly (80.6%) is composed of repeats, notably long terminal repeat retrotransposons and simple repeats; it also has a unique abundance of Copia retrotransposons that expanded ~2.1 million years ago (Jiao *et al.*, 2020), suggesting that it might have experienced a relatively recent genome expansion. In contrast, the first multicellular zygnematophyte genomes, those from the species *Zygnema circumcarinatum*, have small genomes of as little as 67 Mb (Feng *et al.*, 2024). Yet, even within this genus, huge variation occurs. *Zygnema* cf. *cylindricum*, which is closely related to *Zygnema circumcarinatum*, has a genome of ~360 Mb; Čertnerová (2021) even described that some genomes of *Zygnema* spp. have a size of >2.7 Gb. Thus, as expected (although still surprising in its starkness), apparent morphological complexity is not correlated with genome size in streptophyte algae. We thus turn next to the detailed insights into those algal traits that were gleaned from these genomes and are of general interest to the plant evolutionary community.

## TRAIT EVOLUTION: INTRICATE MOLECULAR SIGNALLING PATHWAYS

Instead of attempting to reconstruct ancestral streptophyte organisms in their entirety, we focus on the reconstruction of specific traits of interest. The reconstruction of key traits in hypothetical common ancestors provides important insights into their biology, which, in turn, let us understand the evolution of these traits. The spotlight is often on traits that are deemed decisive for body plans, physiology or habitat preferences, among others.

The foremost sources of novel traits are not structural and functional genes, but rather those underpinning gene regulation. Here, transcription factors have gained major attention. Comparative studies inferred that >80% of the transcription factors present in the common ancestor of land plants were already present in earlier streptophyte algal ancestors (Catarino *et al.*, 2016; Wilhelmsson *et al.*, 2017), indicating that much of the genomic novelty in streptophytes pre-dates the origin of land plants.

Land plant biology is integrated by an intricate balance of phytohormones, recognized by signalling pathways with deep evolutionary roots (Ohtaka *et al.*, 2017; De Clerck *et al.*, 2018; Matthes *et al.*, 2019; Carrillo-Carrasco *et al.*, 2023; Chini *et al.*, 2023; Van de Poel and de Vries, 2023; Schmidt *et al.*, 2024). The presence and distribution of these phytohormones and their signalling throughout the green lineage are recurrent themes in plant evolutionary developmental biology. Many of the phytohormones were once deemed to be land-plant specific or even angiosperm specific. But genomic investigations of understudied land-plant lineages and streptophyte algae have drastically changed that picture. With the *Physcomitrium patens* and *Marchantia polymorpha* becoming model plants with sequenced genomes, it became apparent that most phytohormone biosynthesis and signalling routes are broadly distributed across embryophytes (Rensing *et al.*, 2008; Bowman *et al.*, 2017; Eklund *et al.*, 2018). The first streptophyte algal genome (*Klebsormidium nitens*) already revealed a deeper origin of many phytohormone-relevant genes (Hori *et al.*, 2014), and their expression was quickly found to change upon environmental perturbance in the closely related species *Klebsormidium crenulatum* (Holzinger and Becker, 2015). Schmidt *et al.* (2024) provided a systematic screen of abscisic acid (ABA), auxins, cytokinins (CKs), salicylic acid (SA) and jasmonates across all the main streptophyte algal lineages. Overall, their data suggest that: (1) all types of phytohormones are present in at least one species of the screened streptophyte algae; (2) differences in presence and phytohormone concentration are evident across the different species, even within one streptophyte algal order; and (3) the few embryophytes tested revealed a higher diversity of different auxins and CKs. In streptophyte algae, certain phytohormones were accumulated sometimes only stationarily, possibly in stress conditions. This was mainly apparent in the stress-associated hormones ABA and jasmonic acid (JA). Yet, high amounts of JA are produced mainly by vascular plants (Chini *et al.*, 2023). The gaseous hormone ethylene (ET) was also identified in the Zygnematophyceae alga *Spirogyra pratensis* Transeau (Ju *et al.*, 2015).

Many phytohormones (or subsets/precursors of certain classes of phytohormones) were probably already present in the last common ancestor of streptophytes. In extant species, the amount of phytohormones in comparable tissues varies, often showing lineage-specific profiles (Gachet *et al.*, 2017; Jia *et al.*, 2023; Schmidt *et al.*, 2024). What does this mean for the roles and functions of phytohormones?

### Ethylene

Ethylene was one of the first investigated phytohormones in streptophyte algae. Candidate genes for ET signalling from *Spirogyra* can partly complement *A. thaliana* mutants. Ju *et al.* (2015) found that overexpression of Sp*ETR1* in *etr1-7etr2-3ein4-4* triple mutants of *A. thaliana* resulted in a phenotype similar to *etr2-3ein4-4* double mutants; the double mutant performed slightly better than the triple mutant—but neither the Sp*ETR1* complement nor the double mutant shows a wild-type phenotype. However, it is conceivable that Sp*ETR1* was able to complement the phenotype caused by lack of At*ETR1* in the triple mutant. A similar picture emerged in this study for EIN3, whereby overexpression of Sp*EIN3* on an *Atein3* mutant background is inducible by the ET precursor 1-aminocy-clopropane-1-carboxylic acid and partly rescues the reduction in hypocotyl length upon treatment with 1-aminocy-clopropane-1-carboxylic acid observed for wild-type *Arabidopsis* (Ju *et al.*, 2015). Likewise, *Arabidopsis* plants with *35S-*Sp*EIN3-YFP* on an *ein3* background are rescued regarding the induction of the transcription factor *ERF1* upon ET treatment (Ju *et al.*, 2015). In *Spirogyra* itself, *ERF1* is also induced upon ET treatment, which leads, furthermore, to algal cell elongation and expression of stress-related genes (Ju *et al.*, 2015; Van de Poel *et al.*, 2016).

### Abscisic acid

In comparison to ET, the signalling pathways of phytohormones other than ethylene are more divergent. They are, however, particularly illuminating with respect to understanding how complex cascades might emerge; sometimes through relatively little evolutionary change. Here, ABA is a point in case. Perception of ABA requires a signalling cascade built on perception of ABA by PYRABACTIN RESISTANCE 1-LIKE/REGULATORY, short PYL, followed by a PYL-dependent release of suppression of SNF1-RELATED PROTEIN KINASE 2 (SnRK2) by inhibition of PROTEIN PHOSPHATASE 2C (PP2C) (Ma *et al.*, 2009; Park *et al.*, 2009; Rubio *et al.*, 2009; Cutler *et al.*, 2010; Nishimura *et al.*, 2010). SnRK then activates the downstream responses in ABA signalling (Umezawa *et al.*, 2009; Vlad *et al.*, 2009). PYL diversified in tracheophytes, but homologues are present in Zygnematophyceae (de Vries *et al.*, 2018; Cheng *et al.*, 2019; Sun *et al.*, 2019, Feng *et al.*, 2024). Downstream of PYL, the cascade appears conserved and functional in *Klebsormidium*, suggesting a deep conservation of this signalling pathway (Lind *et al.*, 2015). Nonetheless, the *Zygnema circumcarinatum* PYL homologue is non-perceptive of ABA; instead, it engages in an ABA-independent suppression of PP2C (Sun *et al.*, 2019).

### Gibberellic acid

The controlled perception of gibberellin (GA) via the canonical receptor in angiosperms, GID1, is enzymatically functional only in tracheophytes, and the GA-ligand spectrum is likely to have diversified in the ancestor of seed plants (Yoshida *et al.*, 2018). The modulators of GA response, the DELLA proteins that are recognized and degraded upon GA binding to GID (Murase *et al.*, 2008), are specific to land plants. DELLA itself seems to have an ancient ability to interact with a whole plethora of transcription factors that is conserved in embryophytes (Briones-Moreno *et al.*, 2023). DELLAs function as transcriptional activators by interacting with the Mediator complex subunit MED15, regulating specific responses through transcriptional coactivation, and this mechanism is conserved in land plants (Hernández-García *et al.*, 2024).

### Defence hormones

The evolution of jasmonate signalling involved a switch in ligand preference. *Arabidopsis thaliana* preferentially accepts JA as a ligand for its F-box receptor CORONATINE INSENSITIVE1 (COI1), whereas the liverwort *M. polymorpha* binds dn-OPDA and derivatives thereof to mount a full jasmonate response (Monte *et al.*, 2018; Kneeshaw *et al.*, 2022). Concomitant with the difference in ligand binding rapid induction of JA and dinor-12-oxo-phytodienoic acid (dn-OPDA) after wounding was present in some vascular plants; all lycophytes and most bryophytes induced dn-OPDA and dn-*iso*-OPDA (Chini *et al.*, 2023). Downstream signalling and the responses to stress were well conserved between *M. polymorpha* and *A. thaliana* (Monte *et al.*, 2018).

Another defence-associated phytohormone present in streptophyte algae and land plants is SA, a phenolic compound that is derived from chorismate. SA can be derived from chorismate via isochorismate or by hydroxylating benzoic acid (Widhalm and Dudareva, 2015). *Arabidopsis* appears to use solely the route via isochorismate, whereas other angiosperms also use a benzoic acid-dependent route (Meuwly *et al.*, 1995; Pallas *et al.*, 1996; Coquoz *et al.*, 1998; Shine *et al.*, 2016; Zhang *et al.*, 2021; Wu *et al.*, 2023). It was hypothesized that bryophytes and streptophyte algae are likely to rely on the hydroxylation of benzoic acid to form SA because isochorismate synthase in most of the organisms is fused with MenC/MenD domains, possibly leading to isochorismate being funnelled into the phylloquinone pathway (de Vries *et al.*, 2023; Jia *et al.*, 2023). Further investigation is required to determine whether this is true. Independent of its synthesis, SA signalling is mediated by nonexpresser of PR genes (NPR). In *A. thaliana*, NPR1 acts as a positive regulator, whereas its paralogues NPR3 and NPR4 are negative regulators (Zhang *et al.*, 1999; Ding *et al.*, 2018). However, the *M. polymorpha* NPR candidate resembled NPR3/4 as a negative regulator of immunity but resembled NPR1 regarding temperature stress (Jeon *et al.*, 2023). Additionally, the hornworts *Anthoceros agrestis* and *A. punctatus* do not encode NPR homologues, but only distantly related genes containing BTB/POZ domains (Li *et al.*, 2020*a*). Thus, further investigation is needed to understand the extent of conservation of SA signalling in the green lineage and whether other lineage-specific solutions exist.

### Auxin

Auxins are typically recognized by the F-box protein TIR1, which stems from a gene duplication that also led to COI1 (jasmonate receptor) (Carrillo-Carrasco *et al.*, 2023). This gene duplication and neofunctionalization occurred in the last common ancestor of land plants, but homologues to TIR1/COI1 are present in streptophyte algae (Carrillo-Carrasco *et al.*, 2023). A similar pattern is observed for the downstream signalling pathway via transcriptional regulation of the auxin response (Mutte *et al.*, 2018; Carrillo-Carrasco *et al.*, 2023): The auxin response factors (ARFs) repressor AUX/IAA occurs first in land plants and stems from a duplication event, but homologues are found throughout Charophyceae, Coleochaetophyceae and Zygnematophyceae. C-ARFs first appeared in the ancestor of streptophytes, while homologues of A/B-type ARFs are found only in land plants, Coleochaetophyceae and Zygnematophyceae (Flores-Sandoval *et al.*, 2018; Mutte *et al.*, 2018; Martin-Arevalillo *et al.*, 2019). Distinct A- and B-type ARFs emerged after duplication from the A/B-type precursor in the last common ancestor of land plants (Flores-Sandoval *et al.*, 2018; Mutte *et al.*, 2018; Martin-Arevalillo *et al.*, 2019). Non-transcriptional regulation of auxin is, on the contrary, conserved throughout the green lineage (Carrillo-Carrasco *et al.*, 2023; Kuhn *et al.*, 2024). Yet, despite the presence or diversification of some transcriptional regulatory paths, *Klebsormidium nitens* is capable of responding to exogenously applied auxin derivative indole-3-acetic acid (IAA). Its application promoted cell division and cell elongation (Ohtaka *et al.*, 2017), suggesting that a role for IAA as a developmental regulator is deeply conserved in streptophyte evolution. How auxins are sensed in streptophyte algae remains unclear. A recent study suggests that the *RAV* orthologue of *Klebsormidium nitens*, which codes for the transcription factor *Kn*RAV, activates auxin-inducible genes, possibly by binding directly to their promoter (Tounosu *et al.*, 2023).

### Cytokinin

Most components of the CK signalling network are conserved in streptophytes (Pinto *et al.*, 2023; Powell and Heyl, 2023). This is in agreement with the presence of certain CKs in streptophyte algae (Schmidt *et al.*, 2024). Moreover, AHK homologues and orthologues from the Zygnematophyceae algae *Mougeotia* and *Spirogyra* respond to heat stress at a transcriptional level, suggesting a role in the algal stress response (de Vries *et al.*, 2020). Exogenous application of CKs to these two algae did not alter expression of the AHK candidates in the algae, which is, however, concordant with observations in *A. thaliana* (Brenner and Schmülling, 2015; de Vries *et al.*, 2020). It should also be noted that diverse kinases (and especially Ser/Thr kinases) are not only responsive at the gene expression level to environmental alterations in *Mougeotia* and *Spirogyra* (de Vries *et al.*, 2020; Fürst-Jansen *et al.*, 2022), but are also recovered as a general conserved feature of predicted gene expression-based networks shared across Zygnematophyceae and land plants (Rieseberg *et al.*, 2024).

To understand the role of CKs further in streptophyte algae, Pinto *et al.* (2023) investigated CKs in *Coleochaete scutata*. Here, the alga had only low endogenous levels of CK; levels that were less than those present in the culture medium (Pinto *et al.*, 2023). In comparison, the work by Schmidt *et al.* (2024) found a tendency for a much higher concentration of CKs in *C. scutata.* The data presented by Pinto *et al.* (2023) suggest that CK levels can be altered by the alga based on the environmental setting or growth conditions. Indeed, they showed that CKs are bioactive on *C. scutata*; upon exogenous application of different types of CKs, *C. scutata* showed morphological changes leading to a more structured growth. However, the authors also note that purines, which are used in angiosperms as a negative control in exogenous application experiments, lead to a comparable phenotype. The reason for that is not known, but Pinto *et al.* (2023) formulated three hypotheses. First, CKs could be merely metabolic intermediates and not a specific signal, although they also suggested that the low levels of endogenous CKs would argue against this possibility. Second, the alga could use a signalling pathway that is not only specific to CKs but also accepts compounds with similar structures. This scenario is not unlikely, given that we observe similar situations for other phytohormone pathways. Third, despite the lack of a CK receptor in *C. scutata*, CKs could be perceived via alternative (perhaps less specific) routes.

### Chemodiverse signals beyond the classical hormones

Other signalling molecules that integrate land plant biology and the response to environmental cues are specialized metabolites. The evolution of specialized metabolites is complex. On the one hand, there are many lineage-specific duplications in the genes encoding for enzymes that catalyze the generation of specialized metabolites. On the other hand, the diversity of specialized metabolites is seen through an angiosperm-centric lens, biasing our perception of which compounds are critical to embryophyte biology. Having said that, genomic analyses of diverse specialized metabolism pathways have suggested that many core pathways are conserved to some degree (Dadras *et al.*, 2023*a*; Rieseberg *et al.*, 2023). Some embryophyte genes have orthologues across streptophytes, whereas other genes display lineage-specific radiations of paralogues in streptophyte algae. This was observed particularly for the phenylpropanoid pathway and its routes towards lignin (de Vries *et al.*, 2021). The pathway is modular and includes several promiscuous enzymes, such as CYP450 (Alber *et al.*, 2019; Hansen *et al.*, 2021). Several enzymes in the lignin pathway have been recruited convergently in vascular plants, leading to different pathways in angiosperms and lycophytes (Weng *et al.*, 2008, 2010; Weng and Chapple, 2010; Weng, 2014). This demonstrates that even in the absence of clear orthologues, similar pathways might be possible by convergent recruitment of other gene family members in streptophyte algae, where phenylpropanoid-derived compounds have also been detected. Flavonoids are another interesting case, because their biosynthetic pathway is only conserved in part in land plants (Piatkowski *et al.*, 2020; Davies *et al.*, 2022). Yet, land plants lacking such canonical enzymes produce similar end-products (Gungor *et al.*, 2021). Moreover, divergent pathways to compounds with a similar function, the auronidins and anthocyanins, have been detected in bryophytes (Berland *et al.*, 2019; Davies *et al.*, 2022).

Overall, it is obvious that any organism has small molecules that shape growth and physiology. Why, then, is it particularly promising to study these in streptophyte algae? Here, the conceptual advancement is that an understanding of the action of signalling molecules/phytohormones in algae will, ultimately, allow us to reconstruct via inference what these molecular cascades and pathways might have looked like in the last common ancestor of land plants and algae.

## TRAIT EVOLUTION: CELL DIVISION AND MULTICELLULARITY

Cytokinesis and cell division differ between Chlorophyta and Streptophyta. During cytokinesis, most chlorophytes form a so-called phycoplast, which is a plate of microtubules lying in the plane of division (Fig. 1A). During cell division, the phycoplast is formed by a diaphragm-like ingrowing furrow. This differs from the phragmoplast known from land plants (Gunning and Wick, 1985; Pickett-Heaps *et al.*, 1999; Buschmann and Zachgo, 2016). Among chlorophytes, several variations of a phycoplast exist (see summary by van den Hoek *et al.*, 1988). In contrast to chlorophytes, streptophyte algae evolved different types of cell division, ranging from a simple furrow in *Mesostigma* to the phragmoplast (open mitosis), which is typical for embryophytes (Fig. 1D). The phragmoplast is characterized by the presence of a cytoskeleton, which is arranged parallel to the division plane. In this plane, the cell plate of Golgi-derived vesicles is arranged centrifugally and assembles the new cell wall (Buschmann and Zachgo, 2016; Smertenko, 2018).

The streptophyte algae more divergent from land plants (the KCM grade) possess relatively simple cell-division mechanisms, without forming complex cell walls (Domozych and Bagdan, 2022). Manton and Ettl (1965) demonstrated that *Mesostigma* (Mesostigmatophyceae) divides by furrowing. Cytokinesis in *Chlorokybus* (Chlorokybophyceae) is characterized by a centripetal plasma membrane invagination (cleavage furrow) and the usage of centrosomes during mitotic spindle development (Lokhorst *et al.*, 1988). Cell division in *Klebsormidium* and *Interfilum* (Klebsormidiophyceae) represents an intermediate state between the simple phycoplast and the more complex phragmoplast (Floyd *et al.*, 1972; Pickett-Heaps, 1972, 1975; Lokhorst and Star, 1985). The mitosis is open, with a prominent persistent telophase spindle, which is typical for a phragmoplast in the Phragmoplastophyta, but the cytokinesis is effected by a cleavage furrow typical for algae forming a phycoplast during cell division. Thus, certain characteristics, such as the microtubule arrangement, follow the phragmoplastophytic pattern. In contrast, the Phragmoplastophyta form a cell plate of Golgi vesicles in a phragmoplast during the cytokinesis. The cells of the phragmoplastophytic Charophyceae, Coleochaetophyceae and Embryophyta are connected by plasmodesmata, which has not been reported in Zygnematophyceae, nor in Klebsormidiophyceae (Brunkard and Zambryski, 2017). Lokhorst and Star (1985) described a division of a centripetal cleavage furrow that is impinged on a persistent telophase spindle (Fig. 1B). This type has also been reported for the related taxa *Entransia* and *Hormidiella* (Herburger *et al.*, 2016).

The mechanisms of cell division of the phragmoplastophytic streptophyte algae (ZCC grade) are more complex and characterized by special features shared with land plants (summarized by Pickett-Heaps, 1975; Buschmann and Zachgo, 2016). In fact, this is the origin of the name Phragmoplastophyta. The phragmoplast formed by microtubules persists during the cytokinesis in *Coleochaete* (Coleochaetophyceae) and *Chara* (Charophyceae), and they also form plasmodesmata; the latter are absent in the Zygnematophyceae (Brown *et al.*, 1994; Cook *et al.*, 1997; Cook, 2004*a*; Domozych and Bagdan, 2022). In *Coleochaete*, plasmodesmata are formed by an incomplete fusion of cell plates during early phragmoplast formation (Marchant and Pickett-Heaps, 1973; Fig. 1C). It is not known whether the function of plasmodesmata in these streptophyte algae is similar to those in embryophytes. The cell division in Zygnematophyceae varies from reduced phragmoplast formation, as in the filamentous *Spirogyra* (Pickett‐Heaps and Wetherbee, 1987; Sawitzky and Grolig, 1995), or absent, as in the unicellular *Closterium* (Pickett-Heaps, 1975) or *Zygnema* (Bakker and Lokhorst, 1987). Cytokinesis begins with an ingrowing cleavage furrow and cell-plate formation in the phragmoplast by *Spirogyra* and *Mougeotia* (Fowke and Pickett-Heaps, 1969*a*, *b*; Bech-Hansen and Fowke, 1972; Buschmann and Zachgo, 2016). Unicellular zygnematophytes, such as *Micrasterias*, divide by a cleavage furrow, without participation of a phragmoplast (Kiermayer, 1968; Lütz-Meindl, 2016). However, cell division in Zygnematophyceae has been studied for only a few taxa. The typical cell division with a phragmoplast as known for embryophytes (Fig. 1D) is controlled by a preprophase band (PPB) of microtubules, as seen in liverworts, mosses, hornworts, lycopods, ferns, gymnosperms and angiosperms. In streptophyte algae, a preprophase band can be found only in Zygnematophyceae (Buschmann and Zachgo, 2016).

The origin of multicellularity in Streptophyta is difficult to elucidate because of the lack of a fossil record and owing to the presence of various levels of multicellular complexity (Niklas and Newman, 2020). One important feature for complex body plans is the presence of plasmodesmata, which take part in the cell–cell interactions. As mentioned above, plasmodesmata are present only in the Coleochaetophyceae and Charophyceae. *Coleochaete* forms parenchymateous disc-like thalli, and *Chara* has three-dimensional bodies with complex reproductive organs, such as oogonia and antheridia. The sister group of embryophytes, the Zygnematophyceae, form only simple filaments and special unicells; some of them can be arranged in filamentous colonies (Hall *et al.*, 2008*b*). Hess *et al.* (2022) inferred that the common ancestor of this group could have been secondarily unicellular (simplified from a more complex ancestor) and that the formation of filaments in Zygnematophyceae evolved multiple (up to five) times independently. This complex evolutionary history could be the reason for the absence of plasmodesmata in Zygnematophyceae. In sum, the origin of multicellularity in Streptophyta was accompanied by other innovations that are also typical for embryophytes: complex cell walls, phragmoplasts, plasmodesmata, oogamy, apical growth and the production of phytohormones (Umen, 2014). However, disentangling which features are connected to the origin of multicellularity and which characters relate to terrestrialization remains an unresolved question (see also discussions by Fürst-Jansen *et al.*, 2020). The new phylogenomic framework, coupled with additional genomic and multi-omic investigations are likely to shed more light on the genes that underpin the actualization of multicellularity.

Recurring patterns of filamentous growth being gained and lost (Hess *et al.*, 2022; Bierenbroodspot *et al.*, 2024) have key implications for the dynamics in the evolution of this complex trait. Interestingly, even unicellular green algae possess most of the genes necessary for multicellularity (Prochnik *et al.*, 2010; Umen and Herron, 2021). This suggests that the total loss of these genes does not happen when a lineage evolves secondarily into unicellularity. Consequently, this scenario allows for both forward and backward evolutionary transitions in body plans across diverse clades and extended periods (Hess *et al.*, 2022; Bierenbroodspot *et al.*, 2024). Nonetheless, multicellular bodies have emerged multiple times among streptophytes, suggesting that there could be an ancient set of growth regulators (Bierenbroodspot *et al.*, 2024; Donoghue and Clark, 2024; Feng *et al.*, 2024). Indeed, such regulators are found among streptophyte algae, e.g. RHO of plant (ROP) (Mulvey and Dolan, 2023). This signifies that there is a molecular connection across the likely ~1 billion years of multicellular evolution of streptophytes (Bierenbroodspot *et al.*, 2024; Bowles *et al.*, 2024).

## CONCLUDING REMARKS

Land plants stand out, towering not only physically over most photosynthetic eukaryotes but also in their intricacies: they unite various innovations, and their complex bodies can readily sense environmental cues, process them in internal signalling networks and produce complex responses that integrate growth and stress responses (Scheres and van der Putten, 2017). The flow of auxin comes to mind as a palpable link between molecular action and phenotypic output in shaping a plant body (Friml *et al.*, 2003; Carrillo-Carrasco *et al.*, 2023). Streptophyte algae offer a sampling ground for a diversity of complex trait actualizations, whose study can help us advance our current understanding of the origin of hallmark traits of land plants, but also to discover diversified evolutionary solutions to similar problems. Not only can these traits be catalogued, but also these traits (the phenotype) can be sampled from the living streptophyte algal diversity alongside their molecular pathways (the genotype). These pathways light up the different routes that evolution can take (and has taken) towards diversified body plans, specialized metabolites, cue perception and more. To trace these routes properly and leverage diversity, a solid phylogenetic framework needs to be used. Moreover, accounting for the phylodiversity within each of the six main streptophyte algal lineages is essential to a full understanding of the evolution of complex labile traits, such as secondary metabolism or multicellularity. Given the speed of advancement in finding new major orders of streptophyte algae, it is safe to say that we are not, overall, fully there yet. More phylogenomic investigations of streptophyte algae are bound to uncover new lineages and relationships, demonstrating the astounding diversity that 1 billion years of streptophyte evolution brought forth.

Box 1: **Current taxonomy of streptophyte algae.**

**Class Mesostigmatophyceae Marin & Melkonian, 1999**

Order Mesostigmatales Cavalier-Smith, 1998 emend. Marin and Melkonian, 1999

Family Mesostigmataceae Marin and Melkonian, 1999

**Class Chlorokybophyceae** Irisarri *et al.*, 2021

Order Chlorokybales Irisarri *et al.*, 2021

Family Chlorokybaceae Irisarri *et al.*, 2021

**Class Klebsormidiophyceae** C. Jeffrey ex Guiry, 2023 emend. Bierenbroodspot *et al.*, 2024

Order Klebsormidiales Bierenbroodspot *et al.*, 2024

Family Klebsormidiaceae Bierenbroodspot *et al.*, 2024

Order Entransiales Bierenbroodspot *et al.*, 2024

Family Entransiaceae Bierenbroodspot *et al.*, 2024

Order Hormidiellales Bierenbroodspot *et al.*, 2024

Family Hormidiellaceae Bierenbroodspot *et al.*, 2024

**Class Coleochaetophyceae** Bessey ex Woods, 1894

Order Coleochaetales Bessey, 1907

Family Coleochaetaceae Nägeli,1847

**Class Charophyceae** Rabenhorst, 1863

Order Charales Dumortier, 1829

Family Characeae S.F. Gray, 1821

**Class Zygnematophyceae** Round ex Guiry, 2013

Order Serritaeniales S.Hess & J.de Vries in Hess *et al.*, 2022

Family Serritaeniaceae S.Hess & J.de Vries in Hess *et al.*, 2022

Order Zygnematales Bessey emend. S.Hess & J.de Vries in Hess *et al.*, 2022

Family Zygnemataceae Kützing, 1843*

Order Spirogyrales Clements 1909 emend. S.Hess & J.de Vries in Hess *et al.*, 2022

Family Spirogyraceae Blackman & Tansley, 1902

Order Spirogloeales Melkonian, Gontcharov & Marin in Cheng *et al.*, 2019

Family Spirogloeaceae Melkonian, Gontcharov & Marin in Cheng *et al.*, 2019

Order Desmidiales Bessey, 1907 emend. S.Hess & J.de Vries in Hess *et al.*, 2022

Family Desmidiaceae Ralfs, 1848*

(*polyphyletic, needs revisions)
